# Association between glucose levels at admission and outcomes of pneumonia: a systematic review and meta-analysis

**DOI:** 10.1186/s12890-024-03126-2

**Published:** 2024-07-30

**Authors:** Siqi Yuan, Yixia Chen, Ling Xie

**Affiliations:** Intensive Care Unit, The First People’s Hospital of Linping District, 369 Yingbin Road, Nanyuan Street, Linping District, Hangzhou City, Zhejiang Province 311199 China

**Keywords:** Blood glucose, Diabetes mellitus, Meta-analysis, Pneumonia

## Abstract

**Background:**

Elevated blood glucose at hospital admission is frequently observed and has been associated with adverse outcomes in various patient populations. This meta-analysis sought to consolidate existing evidence to assess the association between elevated blood glucose at admission and clinical outcomes amongst pneumonia patients.

**Methods:**

We searched PubMed, Medline, Cochrane library, Web of Science (WoS), and Scopus databases for studies, published up to 31 August 2023, and reporting on the clinical outcomes and the blood glucose levels at admission. Data were extracted by two independent reviewers. Random-effects meta-analyses were used to pool odds ratios (ORs) with 95% confidence intervals (CI) for dichotomous outcomes and weighted mean differences (WMDs) for continuous outcomes.

**Results:**

A total of 23 studies with 34,000 participants were included. Elevated blood glucose at admission was significantly associated with increased short-term (pooled OR: 2.67; 95%CI: 1.73–4.12) and long-term mortality (pooled OR: 1.70; 95%CI: 1.20–2.42). Patients with raised glucose levels were more likely to require ICU admission (pooled OR: 1.86; 95%CI: 1.31–2.64). Trends also suggested increased risks for hospital readmission and mechanical ventilation, though these were not statistically significant. Elevated blood glucose was linked with approximately 0.72 days longer duration of hospital stay.

**Conclusion:**

Elevated blood glucose level at the time of hospital admission is associated with several adverse clinical outcomes, especially mortality, in patients with pneumonia. These findings underscore the importance of recognizing hyperglycemia as significant prognostic marker in pneumonia patients. Further research is needed to determine whether targeted interventions to control glucose levels can improve these outcomes.

**Supplementary Information:**

The online version contains supplementary material available at 10.1186/s12890-024-03126-2.

## Introduction

Pneumonia, an inflammatory condition primarily affecting the alveoli in the lungs [1], is a leading cause of morbidity and mortality worldwide, especially among children and the elderly [2]. Rapid identification, accurate assessment, and appropriate management of pneumonia are critical to improving patient outcomes [3]. However, comorbidities and other factors might modulate the disease course and treatment responses.

One such factor is the metabolic state of the patient, especially their glucose levels at the time of admission [4]. Numerous studies demonstrated the association of hyperglycaemia with adverse outcomes in various critical illnesses, including myocardial infarction, stroke, and sepsis [5]. Elevated bloodstream glucose levels can compromise the immune response, potentially providing an environment conducive to bacterial proliferation and decreasing the efficacy of phagocytic cells [6].

Stress-induced hyperglycaemia is a recognized physiological response to acute illness.^5^ The underlying mechanism involves the release of counter-regulatory hormones such as cortisol, catecholamines, glucagon, and growth hormone [5, 7]. These hormones stimulate hepatic glucose production and hamper insulin-mediated glucose uptake in peripheral tissues, leading to transient hyperglycemia [5, 7]. While this hyperglycaemic response might be evolutionarily adaptive, providing increased energy substrates during periods of physiological stress, it may also lead to detrimental outcomes in certain disease contexts, including pneumonia.

Several studies have reported a potential link between elevated glucose levels at admission and poor outcomes in patients with pneumonia [8–10]. Hyperglycaemia could exacerbate the inflammatory response in the lungs, compromise the function of immune cells, and possibly provide a more hospitable environment for bacterial replication [4, 6]. Furthermore, elevated glucose levels might impact the efficacy of antimicrobial agents [11].

While the physiological mechanisms linking hyperglycaemia and poor outcomes in pneumonia patients seem plausible, the clinical evidence is still unclear and controversial. Some studies suggest a clear correlation between glucose levels at admission and the severity of pneumonia or mortality [9, 10], while others fail to demonstrate a statistically significant association [8]. These discrepancies could be attributed to differences in study populations, varying definitions of hyperglycaemia, or heterogeneous pneumonia aetiologies and treatments.

Given the potential implications for clinical practice, understanding the relationship between glucose levels at admission and pneumonia outcomes is crucial.

The study objective is as follows: In patients with pneumonia (Population), does elevated blood glucose levels at hospital admission (Exposure) compared to normal or baseline blood glucose levels at hospital admission (Comparison) result in increased short-term and long-term mortality, ICU admission, readmission, need for mechanical ventilation, and length of hospital stay (Outcomes)?

## Methods

### Eligibility criteria

#### Population

The review included studies with participants diagnosed with pneumonia, and considering the association between their glucose levels at admission and various outcomes. We did not restrict by age, gender, ethnicity, or geographic location.

#### Exposure group

This group consisted of patients with pneumonia who had elevated admission glucose levels upon hospital entry.

#### Comparison group

This group included patients with pneumonia who had normal or baseline admission glucose levels upon hospital entry.

#### Outcomes

The primary outcomes of interest were short-term mortality (ranging from in-hospital death to less than 1 year) and long-term mortality (≥ 1 year). Secondary outcomes included readmission rate, ICU admission rate, and the length of hospital stay.

#### Study design

We incorporated randomized controlled trials (RCTs), observational, and cohort studies. Case series, and case reports were excluded. All studies had to be published in English from the inception of searchable databases up to August 2023. Both published works and grey literature were considered, to address potential publication bias.

The methodology employed for this systematic review and meta-analysis adhered stringently to the Preferred Reporting Items for Systematic Reviews and Meta-Analyses (PRISMA) 2020 guidelines [12]. The protocol was registered at PROSPERO, with the number CRD42023466150.

### Information sources

We conducted a systematic search of electronic databases, including PubMed, Medline, Cochrane library, Web of Science (WoS), and Scopus. We searched clinical trial registries, including ClinicalTrials.gov and the World Health Organization’s International Clinical Trials Registry Platform (ICTRP), to identify unpublished or ongoing studies that could provide relevant data. We contacted the authors of included studies and relevant grey literature to request additional data, unpublished results, or clarification on study details. Reference lists of included studies were manually searched for additional relevant studies, including unpublished or grey literature.

We combined terms associated with “Pneumonia,” “Blood Glucose,” and the specific “outcomes” mentioned above, using both Medical Subject Headings (MeSH) and relevant keywords. There were restrictions on English language and publications time (till 31 August 2023).

### Study records

#### Data management

EndNote X9 citation management software was used to organize the retrieved studies, and duplicate entries were removed.

#### Selection process

Two independent researchers meticulously screened the titles and the abstracts of the retrieved studies. Full texts of relevant studies were then assessed for potential inclusion in our review. Cases of disagreement were settled through discussions or by seeking the judgment of a third expert reviewer.

#### Data collection process

An uniformly structured data extraction form was used by two independent reviewers. Details such as study attributes (name of authors, year of publication, research design, and the setting of the study), characteristics of the participants (total count, age distribution, gender ratio, severity of the ailment), specifics of the exposure and the reference groups, and the outcomes that were documented. Furthermore, in the interest of transparency, funding avenues for each study were assessed for any potential conflicts of interest.

### Risk of bias assessment

Risk of bias inherent in the individual studies was assessed independently by two reviewers using the Newcastle Ottawa Scale (NOS) [13]. The NOS employs a star-based system, and studies are judged on three broad domains: selection, comparability, and the ascertainment of the outcome of interest for cohort studies or the exposure of interest for case-control studies. The total highest quality score a study can attain is nine stars, with higher scores indicating a lower risk of bias. For RCTs, Cochrane Risk of bias-2 (RoB-2) tool was used and the study was classified as high risk, low risk or some concerns [14]. Cases of disagreement were settled through discussion or by consulting with the third reviewer.

### Data synthesis

Meta-analyses were performed using STATA version 14.2 software. Due to the anticipated methodological and clinical heterogeneity, a random-effects model was employed [15]. The primary measures of effect were the pooled odds ratio (OR) with 95% confidence interval (CI) for the dichotomous outcomes and the weighted mean difference (WMD) with 95%CI specifically for the continuous outcome (i.e., length of hospital stay).

Forest plots were used to visually convey the data, where individual study effects were denoted by squares (sized in accordance to the study’s weight) and their 95% confidence intervals (CIs) represented by horizontal lines. The collective effect size and its CI were symbolized by a diamond shape located at the base of each forest plot. To determine the extent of heterogeneity, the I^2^ statistic was deployed [15]. Subgroup analyses were conducted based on blood glucose levels and study design to offer a more granular understanding of the associations.

Potential publication bias was assessed for outcomes where at least ten studies were available using funnel plots and Egger’s regression test. Sensitivity analyses were predefined and conducted to assess the robustness of our results. We performed leave-one-out sensitivity analyses, where each study was excluded one at a time to assess its influence on the overall results.

Meta-regression analysis was conducted to explore potential sources of heterogeneity and to examine the influence of various study-level covariates on the association between elevated blood glucose levels at admission and short-term mortality in pneumonia patients. However, meta-regression was possible for only one outcome, short-term mortality, as it was the only outcome with at least 10 studies, meeting the minimum criteria for conducting meta-regression.

## Results

### Search results

**Fig. 1:**
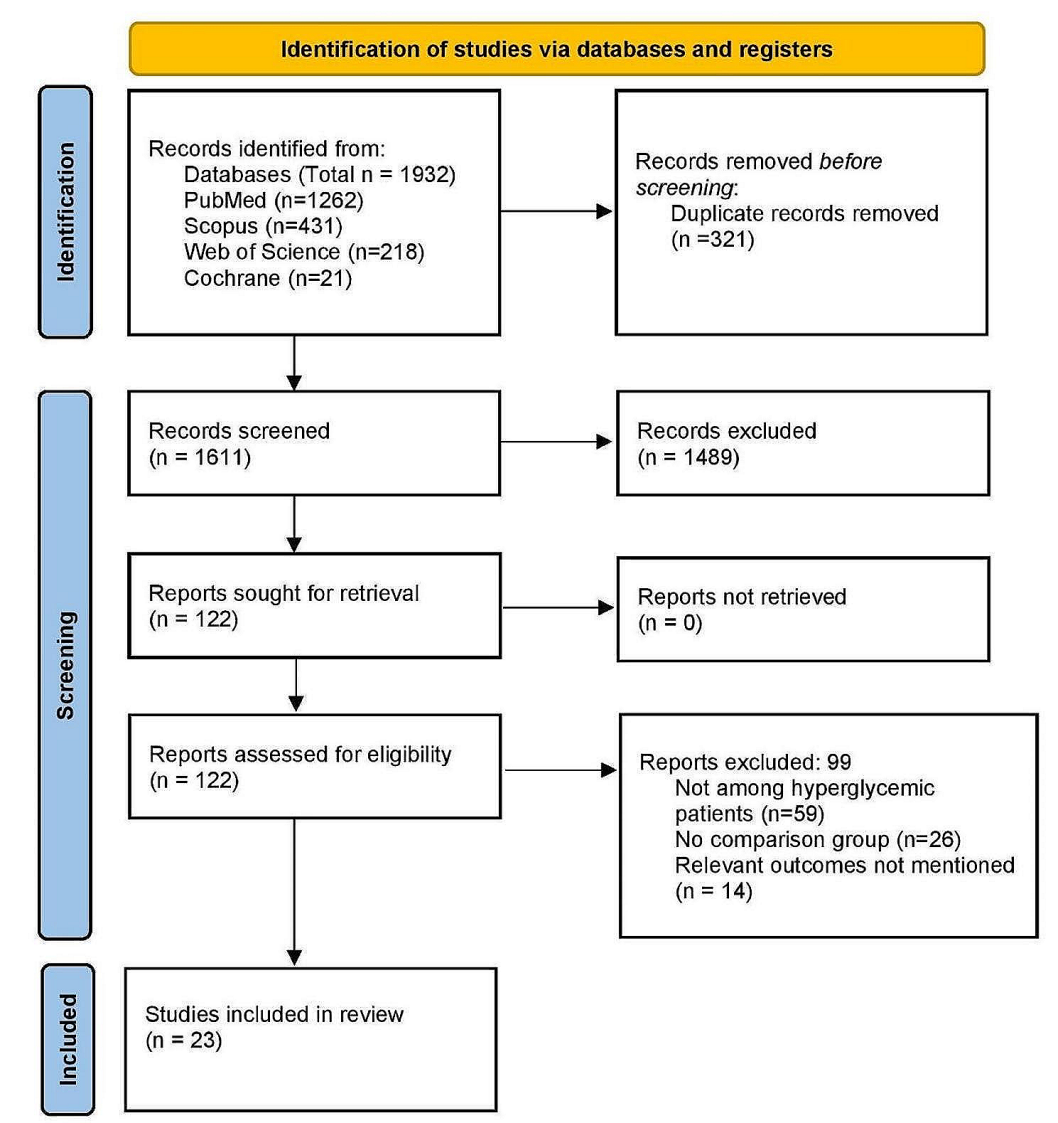
PRISMA flowchart

In primary screening, 1932 citations were identified across the databases. Following duplicates removal, 122 full-text articles were retrieved, and underwent secondary screening. Finally, a total of 23 studies satisfied the eligibility criteria and were included in the analysis (Fig. 1) [8–10, 16–36].

### Characteristics of the included studies

Of 23 studies, a significant portion (*n* = 7) were conducted in the USA, followed by China (*n* = 4). The research designs predominantly comprised retrospective studies (*n* = 10) and prospective studies (*n* = 9). Sample sizes among the studies varied significantly, from as few as 95 to as many as 6891 participants. Most of the studies focused on adults and elderly populations diagnosed with community-acquired pneumonia. The blood glucose cut-off values for assessments were diverse, but 11.1 mmol/L (200 mg/dl) emerged as the most frequent threshold in several studies (*n* = 6). Mortality was the primary outcome in most of the included studies. As shown by the results of the NOS scoring, nine studies had a low risk of bias, nine studies had a high risk of bias, and five studies had a moderate risk of bias (Table 1).

**Table 1:** Study details

Studies	Locations	Study character	Sample size	Participants	Cut-off levels for blood glucose values	Mortality measure (duration)	Method of confounding control	Outcomes assessed	Mean age (in years)	Risk of bias assessment
Akirov 2016	Israel	Prospective	2164	Age ≥65 years with a principal discharge diagnosis of pneumonia	11.1 mmol/L (200 mg/dl)	In-hospital deaths and after 3-years	Regression	Mortality	83	8 (Low)
Bhattacharya 2013	USA	Retrospective	857	Patients aged 40 years and above with admissions for community-acquired pneumonia	7 mmol/L (126 mg/dl)	In-hospital deaths	Regression	Mortality, Readmission, Length of hospital stay	64	5 (Moderate)
Castellanos 2010	USA	Retrospective	387	Age ≥18 years, with pneumonia diagnosed by chest x-ray with symptoms suggestive of pulmonary infection	7 mmol/L (126 mg/dl)	In-hospital deaths	Regression	Mortality, ICU admission, Need for mechanical ventilation	51	5 (Moderate)
Chen 2015	Taiwan	Retrospective	203	All patients with diabetes mellitus admitted for community acquired pneumonia	14 mmol/L (252 mg/dl)	In-hospital deaths	None	Mortality, ICU admission	77.5	2 (High)
Di Yacovo 2013	Spain	Prospective	516	Non-severely immunosuppressed adult patients admitted with pneumonia	11 mmol/L (198 mg/dl)	In-hospital deaths	Regression	Mortality	69	7 (Low)
Eurich 2010	Canada	Prospective	2366	Patients hospitalised with community-acquired pneumonia	7.8 mmol/L (140 mg/dl)	90 days	Regression	Readmission	74	6 (Moderate)
Ferreira 2018	Portugal	Retrospective	224	Patients with previous history of DM and admitted with the diagnosis of community-acquired pneumonia	10 mmol/L (180 mg/dl)	In-hospital deaths	None	Mortality	77	3 (High)
Foltran 2013	Italy	Retrospective	1018	All Patient-Refined Diagnosis-Related Group Code 137 (‘Major Respiratory Infections & Inflammations’) or 139 (‘Other Pneumonia’)	NA	In-hospital deaths	Regression	Mortality	73	3 (High)
Hirata 2013	Japan	Retrospective	185	Adult diabetic patients admitted for the treatment of pneumonia	NA	30 days	Regression	Mortality	75.3	8 (Low)
Iroezindu 2016	Nigeria	Case control study	309	All patients ≥18 years admitted with community-acquired pneumonia	10 mmol/L (180 mg/dl)	In-hospital deaths	Regression	Mortality	55.4	7 (Low)
Jahanihashemi 2018	Iran	Cross-sectional	621	All patients over the age of 12 with community-acquired pneumonia	NA	In-hospital deaths	Regression	Mortality	65	3 (High)
Jensen 2017	Denmark	Retrospective	1318	Hospitalized adult community-acquired pneumonia patients	NA	In-hospital deaths	Regression	Mortality	71	3 (High)
Kinzel 1988	USA	Prospective	95	Patients over the age of 60 admitted for the diagnosis of pneumonia	11.1 mmol/L (200 mg/dl)	In-hospital deaths	None	Mortality	72	3 (High)
Koskela 2014	Finland	Prospective	131	Hospitalised patients who survived at least 30 days after mild-to-moderate community-acquired pneumonia	10.75 mmol/L (194 mg/dl)	5 years	Regression	Mortality, Length of hospital stay	66	5 (Moderate)
Lepper 2012	Switzerland	Prospective	6891	Patients with community acquired pneumonia	14 mmol/L (252 mg/dl)	90 days	Regression	Mortality	59.8	8 (Low)
McAlister 2005	Canada	Prospective	2471	Adults were admitted with a clinical diagnosis of community acquired pneumonia	11 mmol/L (198 mg/dl)	In-hospital deaths	Regression	Mortality, Length of hospital stay	75	5 (Moderate)
Postma 2017	Netherlands	RCT	1549	Aged 18 years or older, admitted with community acquired pneumonia to non-ICU wards, with new pulmonary infiltrate on chest radiography or computed tomography	14 mmol/L (252 mg/dl)	30 days	Regression	Mortality	70	High risk (RoB 2 tool assessment)
Rueda 2010	USA	Prospective	434	Patients for whom a culture of sputum, pleural fluid or blood yielded S. pneumoniae	7 mmol/L (126 mg/dl)	NR	Regression	ICU admission	64.6	3 (High)
Schuetz 2014	Switzerland	Prospective	880	Patients with presumed lower respiratory tract infection presenting to emergency departments	11 mmol/L (198 mg/dl)	30 days	Regression	Mortality	67	3 (High)
Shen 2021	China	Retrospective	1656	Patients with community acquired pneumonia (≥45 years)	11.1 mmol/L (200 mg/dl)	28 days	Regression	Mortality	67.8	8 (Low)
Xu 2023	China	Prospective	250	Viral pneumonia patients	14 mmol/L (252 mg/dl)	90 days	Regression	Mortality	NR	7 (Low)
Yende 2010	USA	Retrospective	1895	Patients hospitalised with community-acquired pneumonia	11.1 mmol/L (200 mg/dl)	Within 1 year	Regression	Mortality	67.2	3 (High)
Zeng 2022	China	Retrospective	290	Patients diagnosed with community-acquired pneumonia	11.1 mmol/L (200 mg/dl)	30 days	Regression	ICU admission, Length of hospital stay, Need for mechanical ventilation	85.6	3 (High)

RCT – Randomized controlled trial; NR – Not reported; NA – Not available; USA – United States of America

### Mortality

#### Short-term mortality

**Fig. 2:**
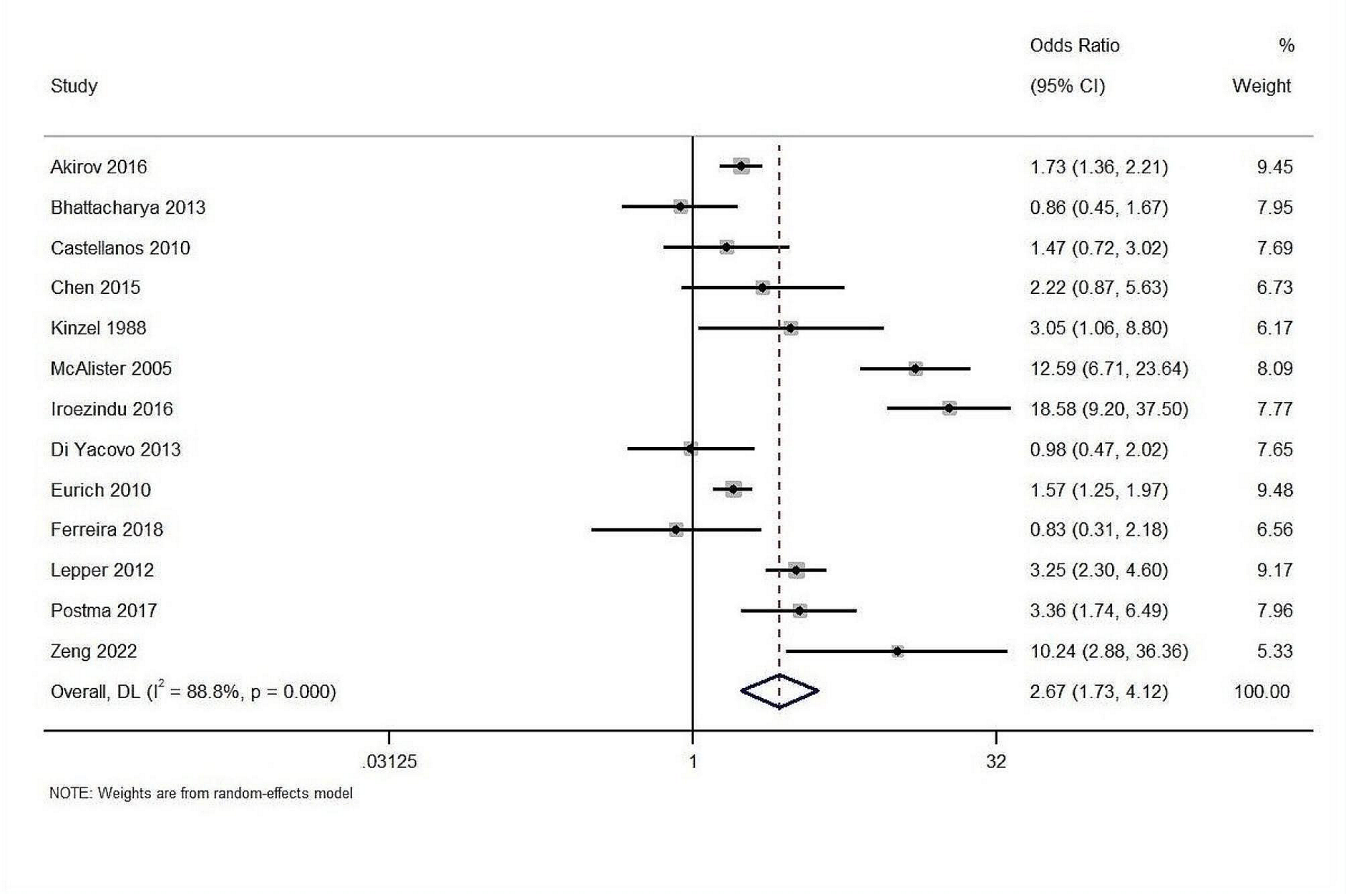
The association between admission glucose levels and short term mortality (analyzed using the number of events)

**Fig. 3:**
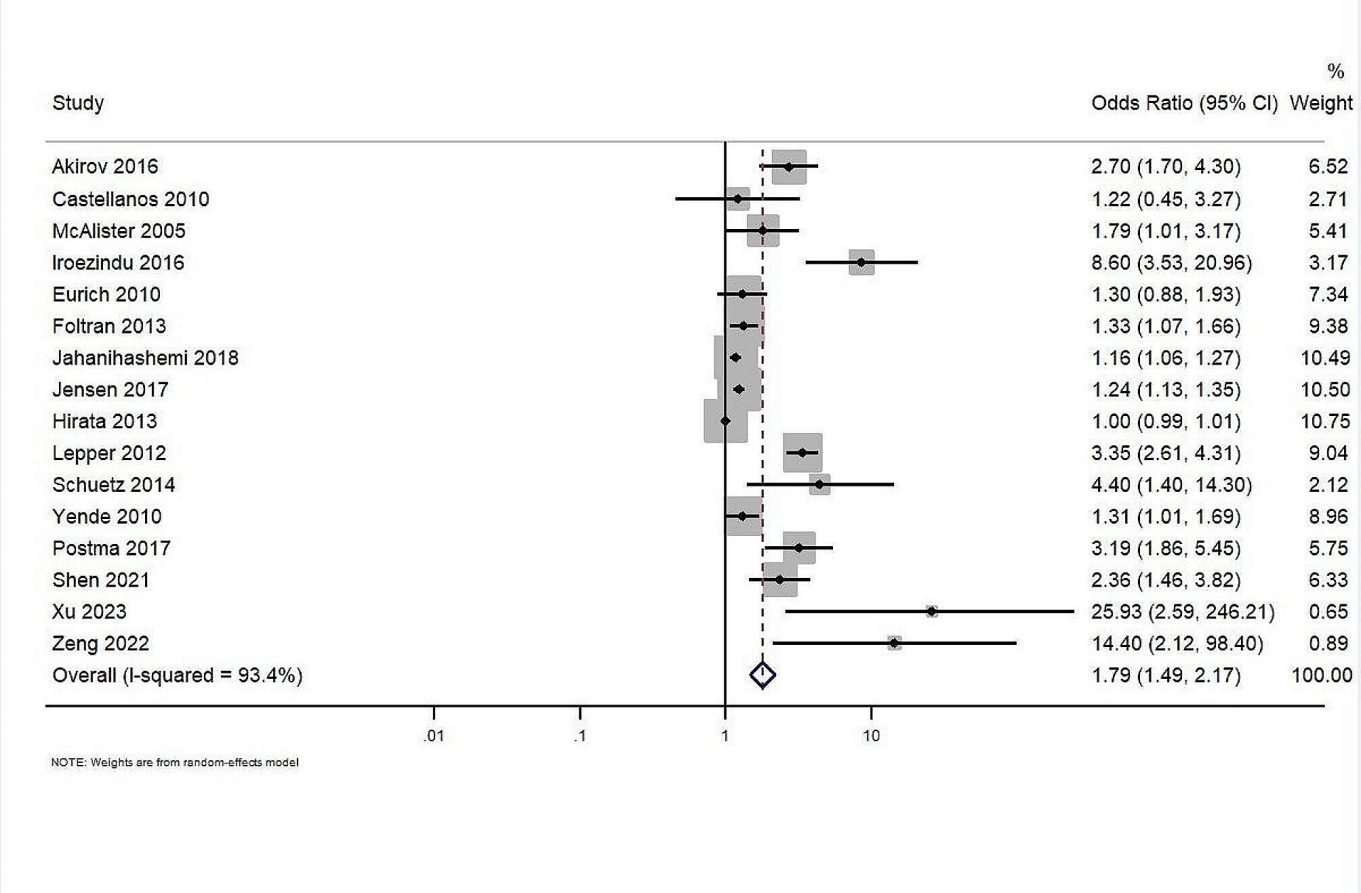
The association between admission glucose levels and short term mortality (analyzed directly using odds ratio)

Thirteen studies with a total of 18,322 participants reported data on mortality. The overall effect estimate demonstrated that patients with altered glucose levels at admission had significantly (2.67-fold) higher risk of mortality (pooled OR: 2.67; 95%CI: 1.73–4.12; z-value of 4.419; *p* < 0.001) (Fig. 2). The Cochran’s Q statistic, calculated for assessing heterogeneity across the studies, was 107.06 (df = 12, *p* < 0.001). This was further quantified by the I² statistic, suggesting that approximately 88.8% of the variability in effect estimates was due to heterogeneity rather than chance and tau² statistic of 0.51. The calculation of effect estimates directly from OR of individual studies also showed a significant association between blood glucose level at admission and short-term mortality (pooled OR = 1.79; 95%CI: 1.49–2.17, *n* = 16, I^2^ = 93.4%) (Fig. 3).

#### Subgroup analysis

Subgroup analysis was done to assess the association between varying cut-off levels of admission blood glucose and mortality outcomes, as summarized in Supplementary Fig. 1.

Five studies reported data of the patients with the cut-off glucose levels of 7.0-10.9 mmol/L (126-196.4 mg/dl) at admission. The pooled estimate showed that patients within this glucose range had an OR of 2.00 (95% CI: 0.78–5.10) for mortality compared to the reference group. Five studies comprised the subgroup with the glucose range of 11.0-13.9 mmol/L (198–250 mg/dl). The pooled OR for mortality within this blood glucose cut-off was 3.50 (95% CI: 1.32–9.30). Three studies reported data on the subgroup of patients with glucose range **≥** 14 mmol/L (252 mg/dl), with a combined OR of 3.15 (95% CI: 2.36–4.22).

Subgroup analysis was also done based on study design (Supplementary Fig. 2). The results indicate that both prospective and retrospective studies show a significant association between elevated blood glucose levels at admission and increased mortality in pneumonia patients. However, the magnitude of this association and the heterogeneity among studies vary between the study designs. Prospective studies demonstrate a stronger and more consistent association (OR = 2.65; 95%CI: 1.66–4.25), whereas retrospective studies exhibit greater variability (OR = 2.71; 95%CI: 0.90–8.21).

#### Publication bias assessment

The obtained slope coefficient of 0.198 (95% CI: -0.695 to 1.090, *p* = 0.636) indicated that the asymmetry detected in the funnel plot might not be due to publication bias (Supplementary Fig. 3). Moreover, the bias coefficient was − 1.053 (95% CI: -4.483 to 2.377, *p* = 0.513), further confirming absence of any significant publication bias in the analysed studies. Sensitivity analysis showed that the pooled estimates were robust to the exclusion of individual studies in the review (Supplementary Fig. 4).

#### Meta-regression

Meta-regression was performed for short-term mortality, which included 13 studies. We examined the influence of study design (prospective vs. retrospective) and the cut-off values for elevated blood glucose levels on the pooled odds ratio for short-term mortality.

Univariable meta-regression analysis was performed to assess the impact of study design (prospective vs. retrospective) on short-term mortality. The results indicated that study design was not a significant moderator of the association between elevated blood glucose levels and short-term mortality. Univariable meta-regression analysis was also conducted to evaluate the effect of different cut-off values for elevated blood glucose levels on short-term mortality. This analysis also revealed that the cut-off values did not significantly affect the association between elevated blood glucose levels and short-term mortality.

Due to the non-significant results in the univariable meta-regression analyses and the limited number of studies, it was not feasible to conduct a multivariable meta-regression analysis.

Although the severity of pneumonia is a critical variable, it was not uniformly reported across the included studies. This lack of consistent reporting precluded its inclusion in subgroup analyses or meta-regression. The heterogeneity in reporting standards for pneumonia severity across studies remains a limitation of this analysis.

#### Long term mortality

**Fig. 4:**
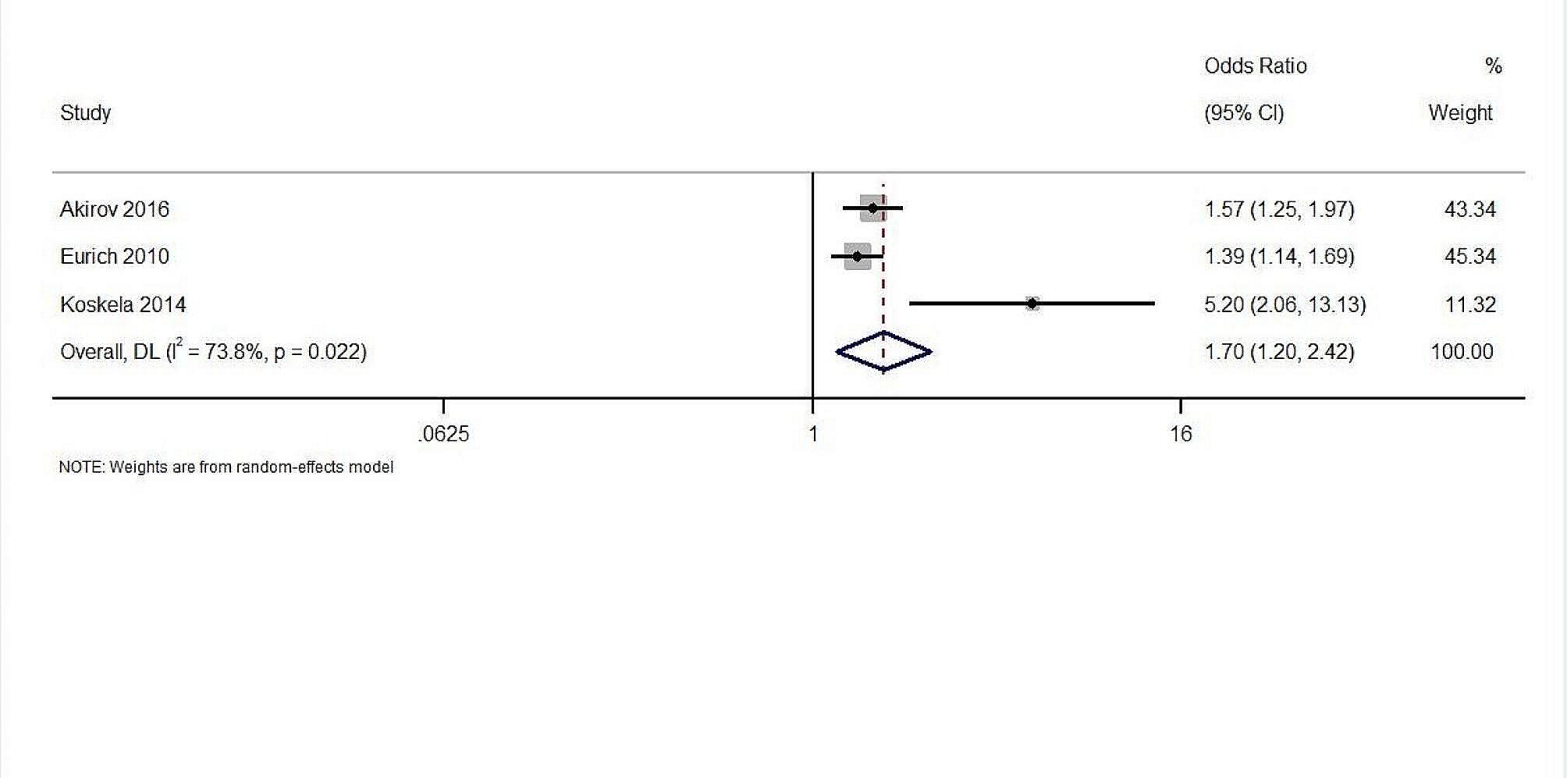
The association between admission glucose levels and long term mortality

Three studies with 4,661 participants reported the outcome of the long-term mortality. Blood glucose levels at admission were associated with a significant rise in long-term mortality, as indicated by the results of the analysis using the random-effects model, with a pooled OR of 1.70 (95% CI: 1.20 to 2.42) (Fig. 4). The z-statistics of 2.973 indicated a statistically significant association with a p-value of 0.003. The observed heterogeneity among these studies, as measured by Cochran’s Q, was statistically significant at 7.63 (df = 2, *p* = 0.022). The I² statistic of 73.8% and and tau² statistic of 0.06 suggested a moderate to high level of heterogeneity.

#### ICU admission

The need for ICU admission was reported in four studies, incorporating a total of 1,428 participants. Our analysis revealed a significant association between admission blood glucose levels and ICU admissions. The pooled OR, derived from a random-effects model, was 1.86 (95% CI: 1.31 to 2.64), with the z-statistic of 3.448 suggesting a statistically significant relationship at a p-value of 0.001 (Supplementary Fig. 5). The Cochran’s Q was 4.59 (df = 3, *p* = 0.204), suggestive of the lack of statistically significant heterogeneity among the studies. However, the I² statistic of 34.7% and tau² statistic of 0.04 suggests a low to moderate level of variation across the studies.

#### Readmission

The risk of hospital readmission was examined in two studies, with a sample size of 3,127 participants. Our meta-analysis showed a pooled OR of 0.83 (95% CI: 0.47 to 1.47; tau² statistic = 0.14) for the association between admission blood glucose levels and hospital readmissions (Supplementary Fig. 6).

#### Requirement of mechanical ventilation

The requirement for mechanical ventilation was explored in two studies, with 791 participants. Our pooled results showed an OR of 1.88 (95% CI: 0.42 to 8.34; tau² statistic = 1.04), suggesting a potential association between elevated admission blood glucose levels and the subsequent need for mechanical ventilation (Supplementary Fig. 7).

#### Length of hospital stay

The length of hospital stay was reported in four studies, involving a total of 3632 participants. Pooling the data using a random-effects model showed that higher blood glucose levels at admission are associated with a slightly higher length of hospital stay, with a WMD of 0.72 days (95% CI: -0.14 to 1.58; tau² statistic = 0.71) (Supplementary Fig. 8).

## Discussion

This study demonstrated that elevated blood glucose level at the time of hospital admission is associated with increased risk adverse outcomes such as mortality, ICU admission, and the possible extended length of hospital stay compared to patients with normal glucose levels.

The relationship between blood glucose levels at admission and a range of adverse clinical outcomes has been an area of interest for clinicians. This systematic review and meta-analysis, encompassing a diverse range of studies, provides a comprehensive evaluation of this relationship, revealing critical insights that may influence clinical management and future research.

Our results show an apparent link between elevated blood glucose levels at admission and increased short-term mortality. This association persisted even when studies were stratified based on varying cut-off levels of blood glucose, although the strength of the relationship varied across these subgroups. While previous individual studies have reported on this relationship [9, 10, 23–26], our review offers a robust synthesis of available evidence, drawing from 13 studies and over 18,000 participants.

Our results demonstrated a clear association between blood glucose levels at admission and a long-term mortality. The derived pooled odds ratio, based on data from three studies with over 4,600 participants, indicated that elevated admission blood glucose was linked with a substantial increase in long-term mortality. This further underscores the enduring consequences of hyperglycemia at admission, extending beyond the immediate clinical scenario.

While the extended length of hospital stay, associated with higher glucose levels at admission, was not statistically significant in our analysis, it still raises valid questions regarding the potential burden on the healthcare resources. An extended hospital stay carries the increased risk of hospital-acquired infections and is associated with higher healthcare costs [36, 37]. Hospital readmissions not only signify potential clinical complications but also represent a significant cost burden for healthcare systems [38]. Our review demonstrated a trend towards increased readmission rates in patients with elevated glucose levels at admission. Though the relationship was not statistically significant in our pooled analysis, the trend warrants further exploration, especially given the implications for patient care and healthcare resource utilization. This makes our findings particularly relevant for healthcare administrators and policymakers.

Our study demonstrated a clear association between elevated blood glucose levels at admission and higher need for ICU admission, which serves as a direct marker of disease severity and clinical deterioration. This observation reinforces the broader narrative of hyperglycemia as a predictor of adverse clinical outcomes. Our results highlight the need for higher vigilance and early interventions in patients presenting with elevated glucose levels.

Previous systematic reviews and meta-analyses have investigated the relationship between hyperglycaemia at admission and adverse outcomes in various clinical scenarios [39]. Our findings align with these studies, further demonstrating that elevated glucose levels, even in the absence of a formal diagnosis of diabetes, can have deleterious effects on clinical outcomes. The consistency of these findings, even across different clinical populations and settings, underscores the potential universal nature of this relationship.

The observed association may be explained by several possible physiological mechanisms. Elevated glucose levels can lead to increased oxidative stress and inflammation, resulting in complications such as cardiovascular events or sepsis [40]. Additionally, hyperglycaemia may result in endothelial dysfunction and coagulation abnormalities, further predisposing the patient to adverse outcomes [41]. It’s also worth noting that elevated blood glucose levels at admission might not just reflect a transient stress response but also indicate previously undiagnosed metabolic dysregulation or diabetes, conditions inherently associated with worse clinical outcomes [42].

The main strength of our study is the comprehensive nature of the literature search, the meticulous methodology adhering to PRISMA guidelines, and the rigorous statistical methods, leading to the robustness to our findings. The inclusion of both published and grey literature minimizes the potential for publication bias, as evidenced by our Egger’s test results.

Our study has some limitations. The marked heterogeneity observed across some outcomes suggests variability in study methodologies, populations, or other unmeasured factors. While we utilized a random-effects model and subgroup analysis to account for this variability, and to explore its potential sources, there is still a chance of some residual unexplained heterogeneity. Additionally, our study does not adjust for potential confounders like age, gender, or co-morbidities which might not have been adequately controlled for in the individual studies. Additionally, only English-language studies were included, which might introduce a language bias.

## Implications for clinical practice

The findings of this meta-analysis have significant implications for clinical practice:

### Early recognition and monitoring

Elevated blood glucose levels at the time of hospital admission should be recognized as a significant prognostic marker in pneumonia patients. Routine glucose monitoring at admission can help identify patients at higher risk of adverse outcomes, enabling clinicians to prioritize these patients for more intensive monitoring and care.

### Targeted interventions

While our study did not directly assess the impact of glucose-lowering interventions, the strong association between hyperglycemia and adverse outcomes suggests that early and targeted interventions to manage blood glucose levels might improve patient outcomes. Clinical trials are needed to evaluate the effectiveness of such interventions in pneumonia patients.

### Multidisciplinary approach

Managing hyperglycemia in pneumonia patients may require a multidisciplinary approach, involving endocrinologists, pulmonologists, and critical care specialists. This collaborative approach can help develop comprehensive care plans that address both the infection and metabolic dysregulation.

### Healthcare resource utilization

Recognizing the potential for longer hospital stays and increased ICU admissions among hyperglycemic patients can help healthcare administrators plan and allocate resources more effectively. Early interventions to control glucose levels might reduce the length of hospital stay and prevent ICU admissions, thereby optimizing healthcare resource utilization.

### Patient education

Educating patients about the importance of blood glucose control, even in the absence of diabetes, can enhance self-management and adherence to treatment plans, potentially improving long-term outcomes.

Future studies might focus on understanding the exact causal relationship between elevated blood glucose and adverse outcomes. Randomized controlled trials are needed to examine the effectiveness of targeted interventions for such patients at the time of admission. Furthermore, understanding the optimal range of glucose levels, especially in critically ill patients, can guide clinical management.

## Conclusion

Our systematic review and meta-analysis reaffirm the association between elevated admission blood glucose levels and adverse clinical outcomes, such as short- and long-term mortality and the need for ICU admission. This review underscores the need for heightened clinical awareness and potential early interventions, offering a step towards better patient outcomes and optimized resource utilization.

### Electronic supplementary material

Below is the link to the electronic supplementary material.

Supplementary Material 1

## Data Availability

The datasets generated and/or analysed during the current study are available in the PubMed, Medline, Cochrane library, Web of Science (WoS), and Scopus.
