# Supplementary material for: Association between glucose levels at admission and outcomes of pneumonia: a systematic review and meta-analysis
Source: BMC Pulm Med. 2024 Jul 30;24:369. doi: 10.1186/s12890-024-03126-2 (PMC11290157; doi:10.1186/s12890-024-03126-2)

Supplementary Figure 1: Subgroup analysis for association between admission glucose levels at various cut-offs and short term mortality


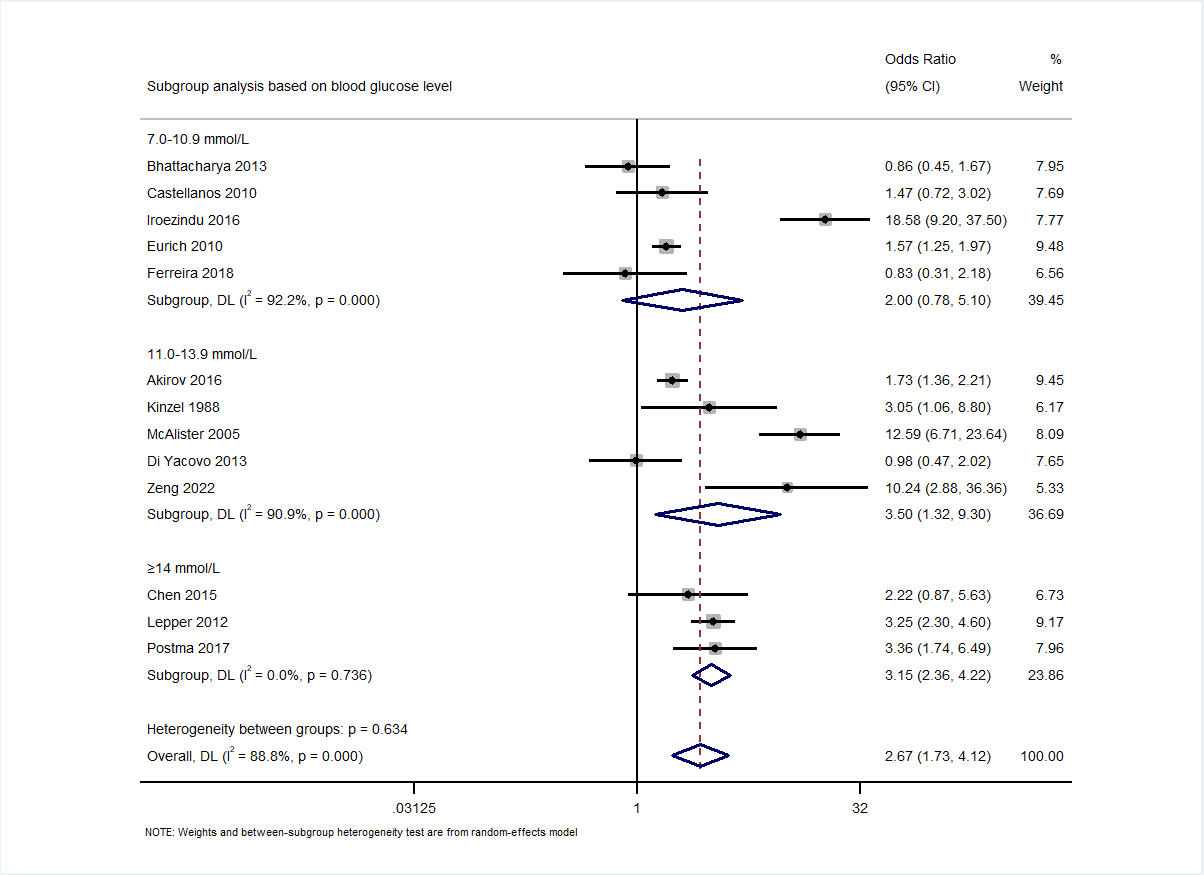


Supplementary Figure 2: Subgroup analysis for association between admission glucose levels and short term mortality stratified by study design


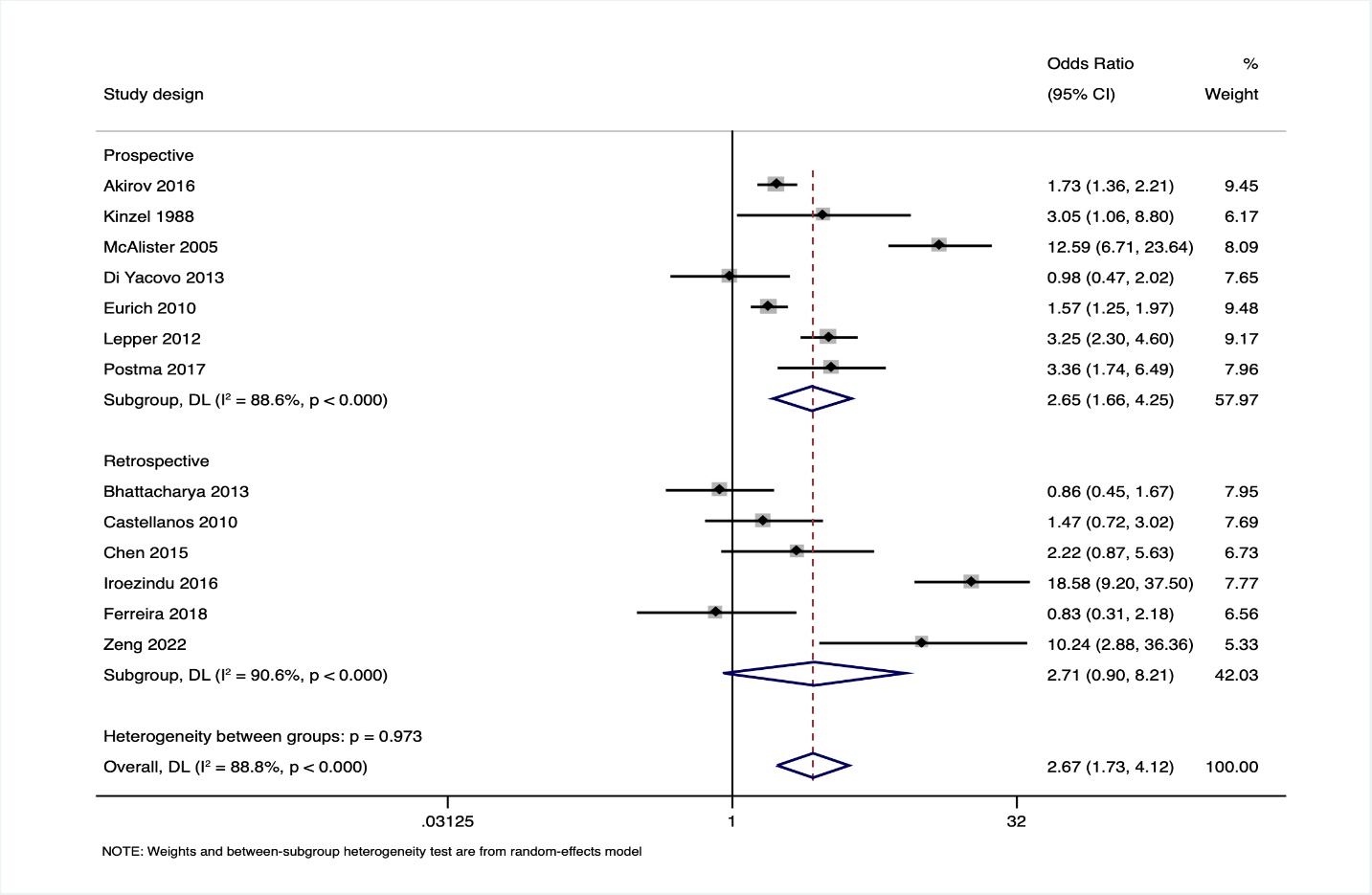


Supplementary Figure 3: Funnel plot for short term mortality


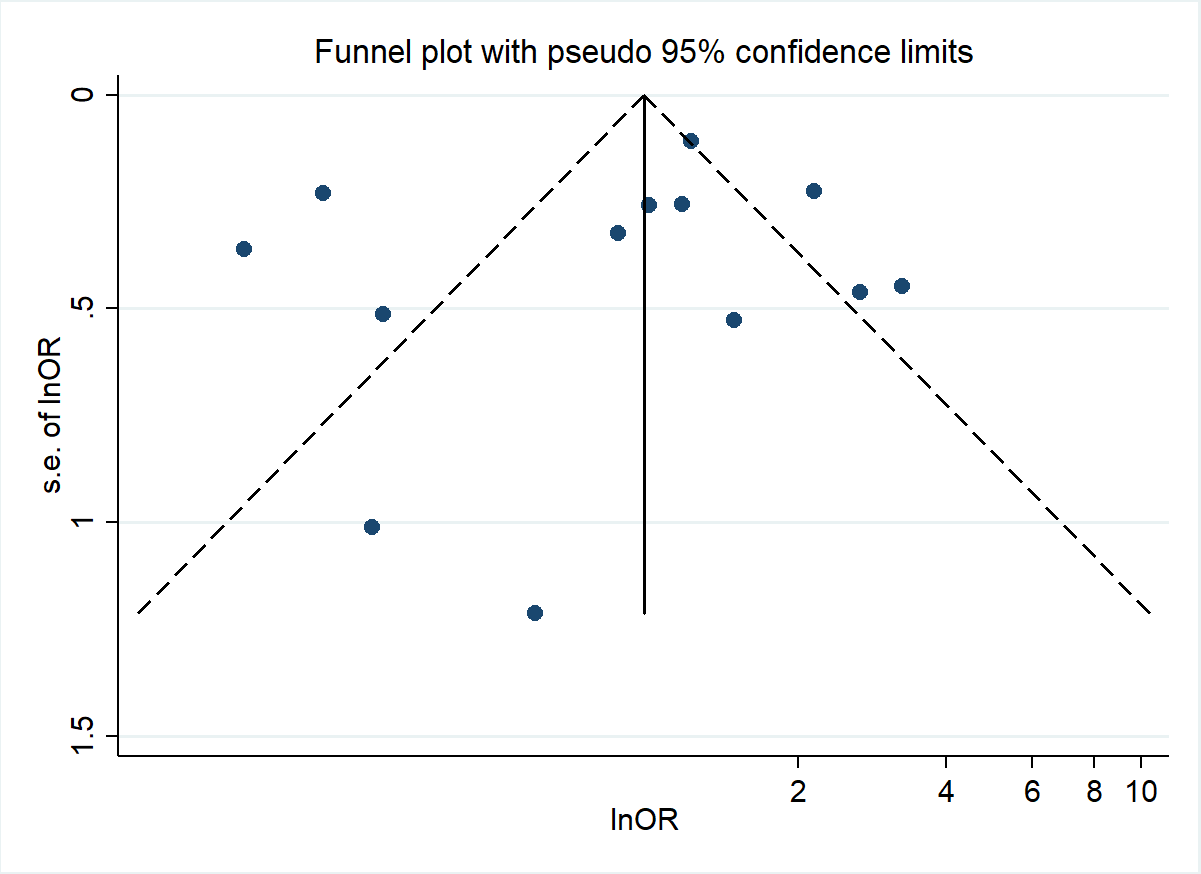


Supplementary Figure 4: Sensitivity analysis results


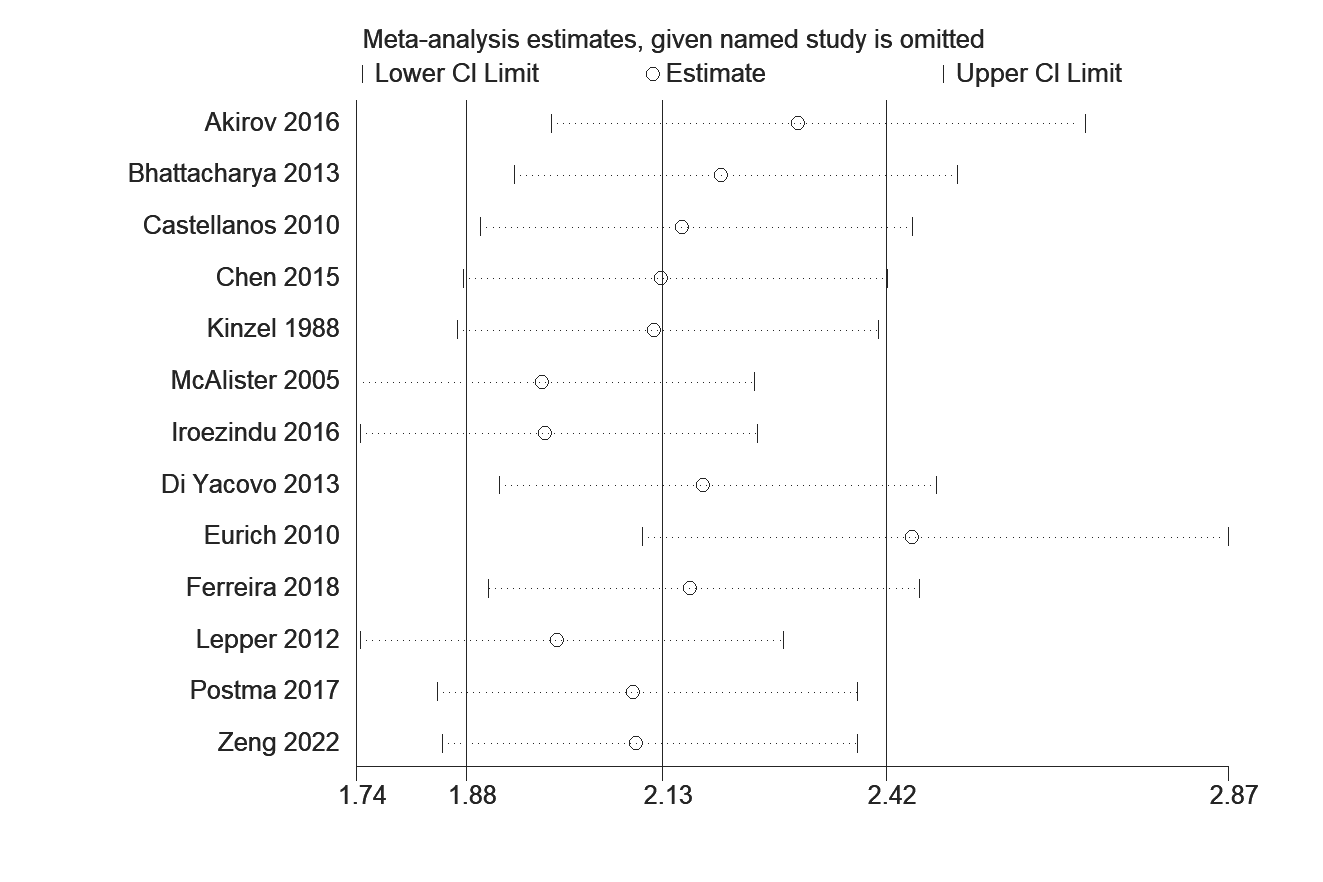


Supplementary Figure 5: The association between admission glucose levels and ICU admission


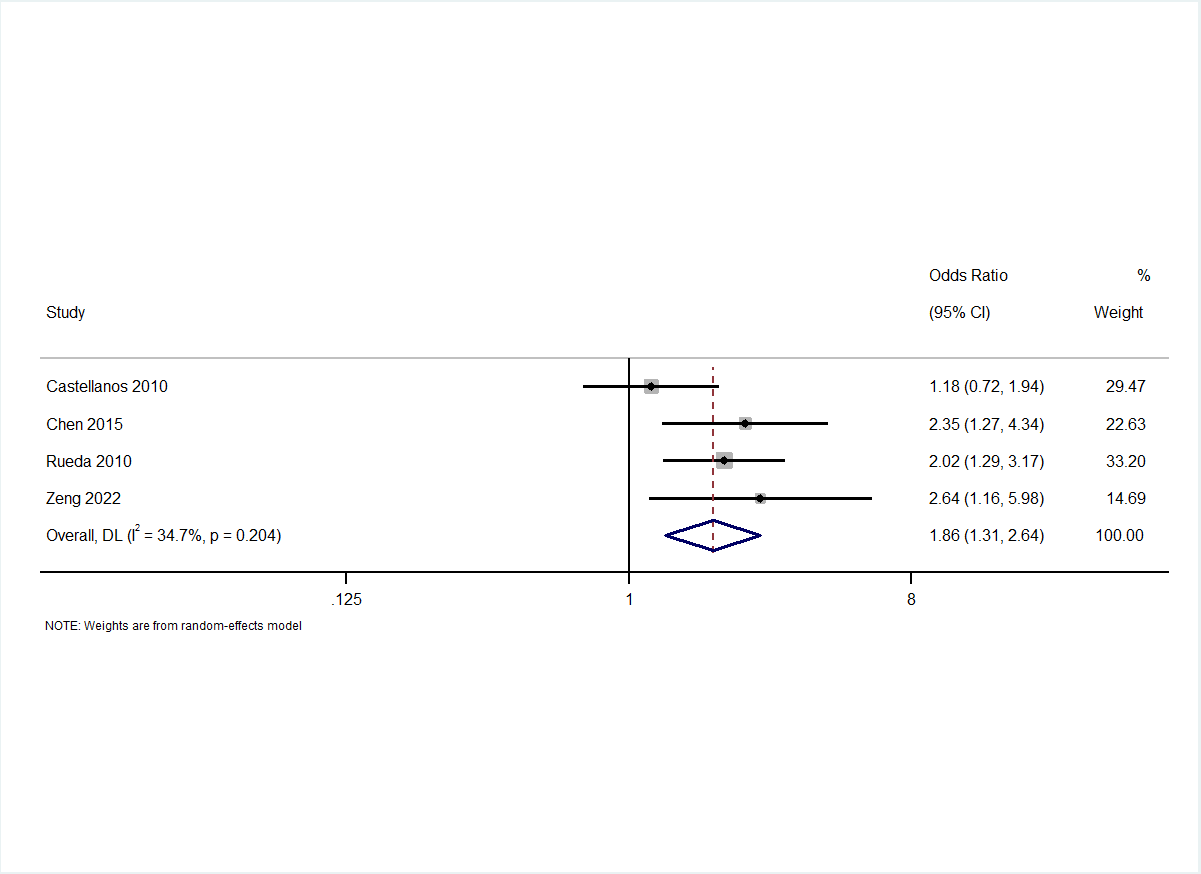


Supplementary Figure 6: The association between admission glucose levels and readmissions


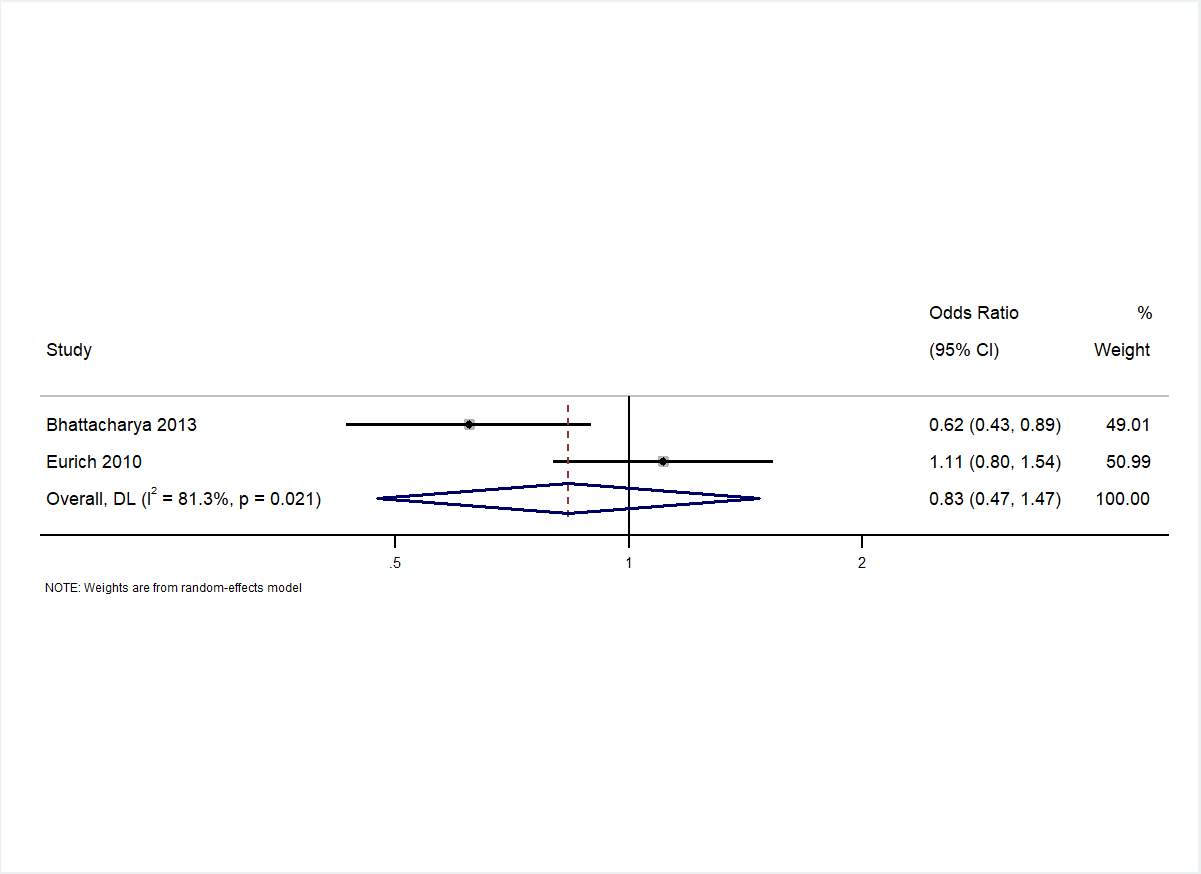


Supplementary Figure 7: The association between admission glucose levels and requirement of mechanical ventilation


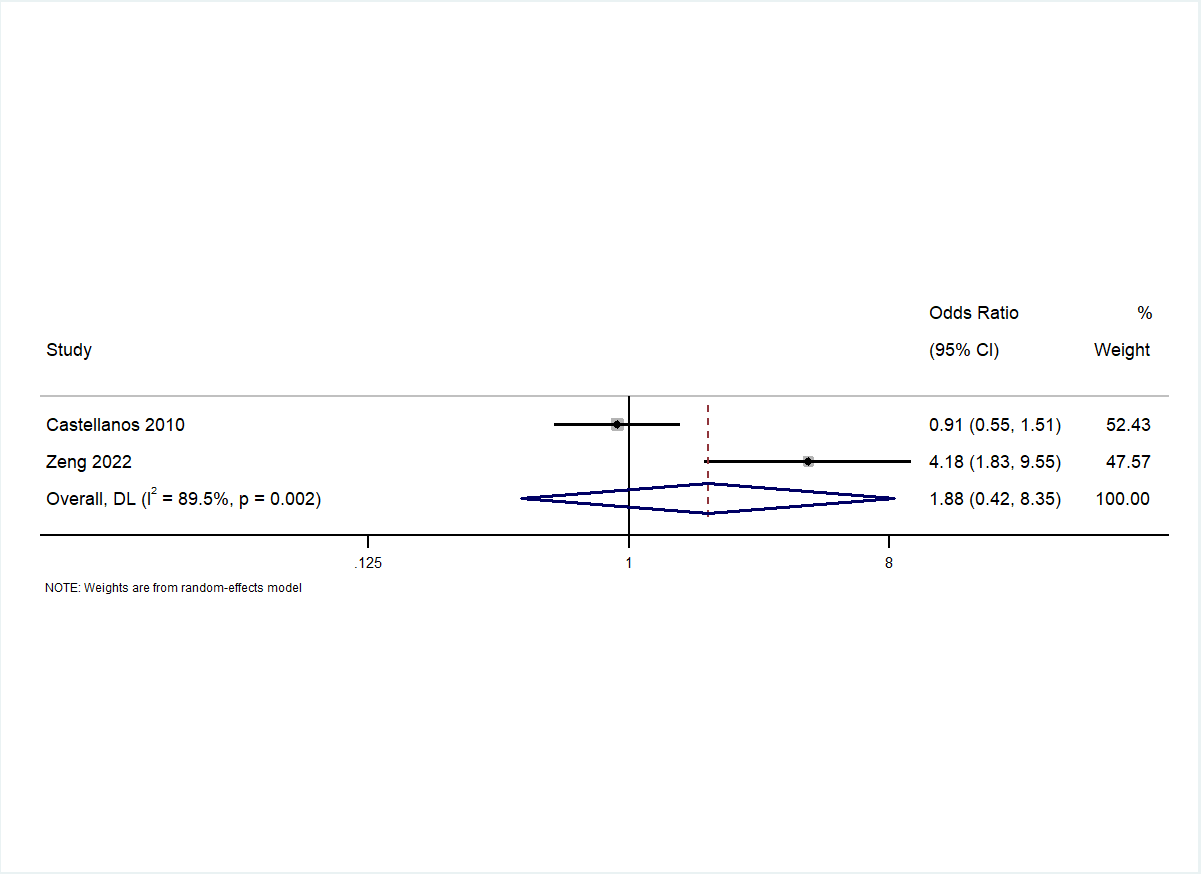


Supplementary Figure 8: The association between admission glucose levels and length of hospital stay


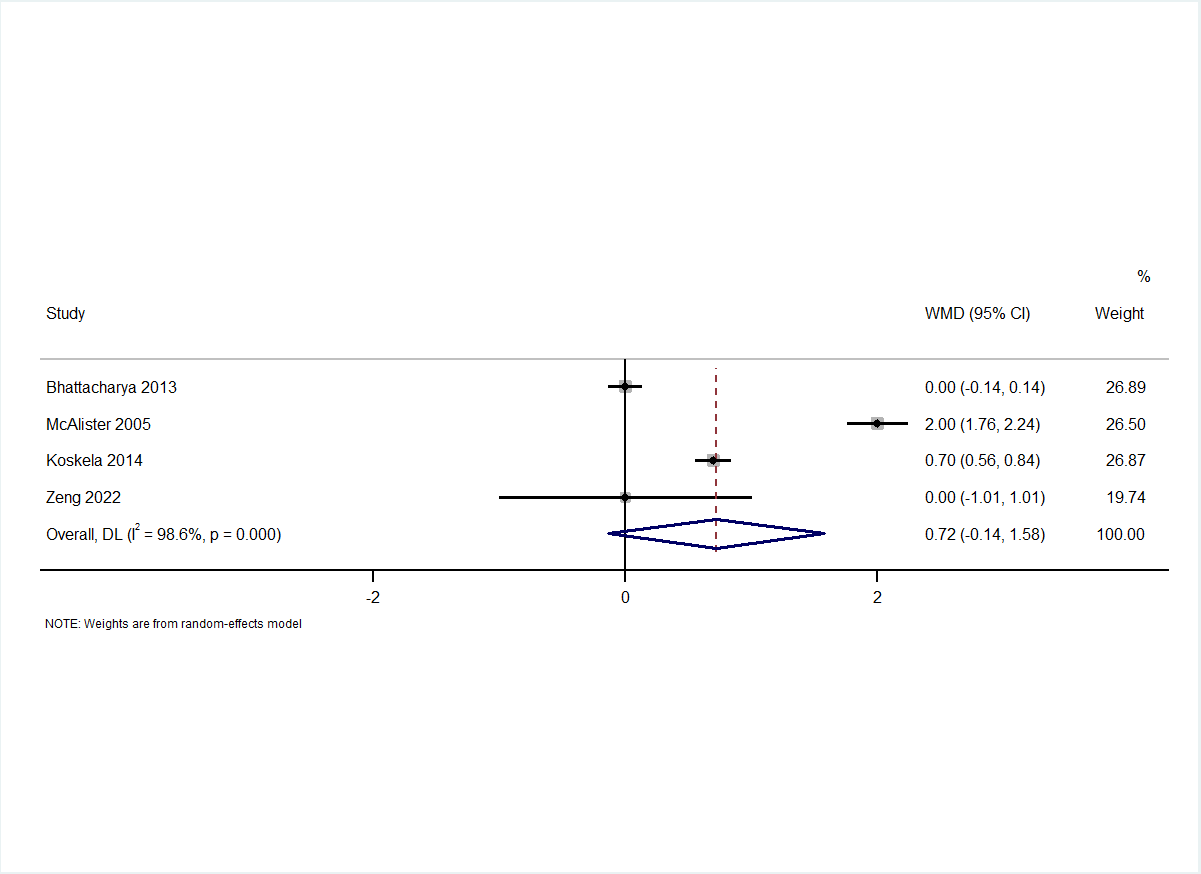

Supplement: Supplementary file 1 — Supplementary Material 1 [file 12890_2024_3126_MOESM1_ESM.docx]
